# Ionotropic GABA and glycine receptor subunit composition in human pluripotent stem cell-derived excitatory cortical neurones

**DOI:** 10.1113/jphysiol.2014.278994

**Published:** 2014-08-28

**Authors:** Owain T James, Matthew R Livesey, Jing Qiu, Owen Dando, Bilada Bilican, Ghazal Haghi, Rinku Rajan, Karen Burr, Giles E Hardingham, Siddharthan Chandran, Peter C Kind, David J A Wyllie

**Affiliations:** 1 Centre for Integrative Physiology, University of EdinburghEdinburgh, EH8 9XD, UK; 2 Centre for Brain Development and Repair, Institute for Stem Cell Biology and Regenerative Medicine, National Centre for Biological SciencesBangalore, 560065, India; 3 Euan MacDonald Centre for MND Research, University of EdinburghEdinburgh, EH16 4SB, UK; 4 Patrick Wild Centre, University of EdinburghEdinburgh, EH8 9XD, UK; 5 Centre for Clinical Brain Sciences, University of EdinburghEdinburgh, EH16 4SB, UK; 6 MRC Centre for Regenerative Medicine, University of EdinburghEdinburgh, EH16 4SB, UK

## Abstract

We have assessed, using whole-cell patch-clamp recording and RNA-sequencing (RNA-seq), the properties and composition of GABA_A_ receptors (GABA_A_Rs) and strychnine-sensitive glycine receptors (GlyRs) expressed by excitatory cortical neurons derived from human embryonic stem cells (hECNs). The agonists GABA and muscimol gave EC_50_ values of 278 μm and 182 μm, respectively, and the presence of a GABA_A_R population displaying low agonist potencies is supported by strong RNA-seq signals for α2 and α3 subunits. GABA_A_R-mediated currents, evoked by EC_50_ concentrations of GABA, were blocked by bicuculline and picrotoxin with IC_50_ values of 2.7 and 5.1 μm, respectively. hECN GABA_A_Rs are predominantly γ subunit-containing as assessed by the sensitivity of GABA-evoked currents to diazepam and insensitivity to Zn^2+^, together with the weak direct agonist action of gaboxadol; RNA-seq indicated a predominant expression of the γ2 subunit. Potentiation of GABA-evoked currents by propofol and etomidate and the lack of inhibition of currents by salicylidine salycylhydrazide (SCS) indicate expression of the β2 or β3 subunit, with RNA-seq analysis indicating strong expression of β3 in hECN GABA_A_Rs. Taken together our data support the notion that hECN GABA_A_Rs have an α2/3β3γ2 subunit composition – a composition that also predominates in immature rodent cortex. GlyRs expressed by hECNs were activated by glycine with an EC_50_ of 167 μm. Glycine-evoked (500 μm) currents were blocked by strychnine (IC_50_ = 630 nm) and picrotoxin (IC_50_ = 197 μm), where the latter is suggestive of a population of heteromeric receptors. RNA-seq indicates GlyRs are likely to be composed of α2 and β subunits.

**Key points**

- This study reports a functional assessment of the subunit composition of inhibitory ionotropic GABA_A_ receptors (GABA_A_Rs) and glycine receptors (GlyRs) expressed by excitatory cortical neurones derived from human embryonic stem cells (hECNs).
- GABA_A_Rs expressed by hECNs are predominantly composed of α2/3β3γ2 subunits; such a composition is typical of that reported for GABA_A_Rs expressed in rodent embryonic cortex.
- Analysis of GlyRs expressed by hECNs indicates they are likely to contain α2 and β subunits – a composition in rodents that is associated with a late embryonic/early postnatal period of development.

## Introduction

γ-Aminobutyric acid (GABA) type A receptors (GABA_A_Rs) are the principal inhibitory neurotransmitter receptors in the mammalian adult brain. GABA_A_Rs are a pentameric ligand-gated anion channels that can be potentially composed of 19 known subunits (α1–6, β1–3, γ1–3, δ, ε, π, θ and ρ1–3), giving rise to a large number of potential receptor stoichiometries (Olsen & Sieghart, 2009). Alongside GABA_A_Rs, strychnine-sensitive glycine receptors (GlyRs) form another major class of pentameric ligand-gated anion channel that can be potentially composed of 5 subunits, α1–4 and β (Lynch, 2009). GABA_A_R and GlyR subunits are each associated with a high degree of spatial and developmental regulation within the CNS (Malosio *et al*. 1991; Laurie *et al*. 1992; Fritschy *et al*. 1994; Flint *et al*. 1998). In this regard, GABA_A_R composition is currently limited to approximately 30 known variants. Moreover, subunit identity typically imparts various pharmacological specificities to the GABA_A_R complex and, collectively, these properties make GABA_A_Rs a key pharmacological target for a range of neurological disorders (Olsen & Sieghart, 2009). The increasing knowledge regarding the functions of GlyRs within the developing CNS indicates that these receptors too are likely to be relevant pharmacological targets (Avila *et al*. 2013a).

The technological advance in the ability to generate human excitatory cortical neurones (hECNs) from pluripotent stem cells (hPSCs) gives the potential to study human-specific physiology and disease *in vitro*. We have previously reported a protocol that generates cultures of predominantly hECNs by 4 weeks of differentiation from anterior neural precursors derived from various stem cell lines (Bilican *et al*. 2014). The translational impact of this technology is ultimately determined by the ability of hECNs to display properties that reflect neurones in their native environment (Yang *et al*. 2011; Sandoe & Eggan, 2013). Indeed, we have previously identified that hECNs are a useful model to study the maturation of AMPAR composition and the reduction in intracellular Cl^−^ concentration that is observed in native neuronal development (Livesey *et al*. 2014). The present study characterises the likely subunit composition of GABA_A_R and GlyRs expressed by hECNs and illustrates that their subunit composition are likely to be similar to those that have been described for inhibitory ionotropic receptors expressed in immature rodent cortex.

## Methods

### *In vitro* hECN preparation

A detailed description of the derivation of hECNs can be found in Bilican *et al*. (2014). Briefly, hECNs were differentiated from anterior neural precursors that were derived from the H9 human embryonic stem cell line (WiCell), which was obtained under ethical/IRB approval of the University of Edinburgh. Experiments were carried out on cells that had been differentiated and maintained in culture for 28–42 days *in vitro* (DIV), or 49–56 DIV. At these time points, around 70% of cells were neuronal (β3-tubulin^+^), with little contamination from neural precursor cells (nestin^+^), astrocytes (GFAP^+^) or GABA-ergic (GAD65/67^+^) interneurons (Bilican *et al*. 2014; Livesey *et al*. 2014). Neurones were consistent with an excitatory (VGLUT1^+^) identity that also exhibited properties of neurones of the upper and lower layers of the cortex (see Bilican *et al*. 2014; Livesey *et al*. 2014).

### Electrophysiology

The whole-cell patch-clamp configuration was used to record currents from hECNs using an Axon Multiclamp 700B amplifier (Molecular Devices, Sunnyvale, CA, USA). Patch electrodes (∼4–7 MΩ) were filled with an ‘internal’ recording solution comprising (in mm): potassium gluconate 155, MgCl_2_ 2, Na-HEPES 10, Na-PiCreatine 10, Mg_2_-ATP 2 and Na_3_-GTP 0.3, pH 7.3 (300 mOsmol l^–1^). Coverslips containing hECNs were placed in the recording chamber, which was superfused with an ‘external’ recording solution composed of (in mm) NaCl 152, KCl 2.8, HEPES 10, CaCl_2_ 2, glucose 10, pH 7.3 (320–330 mOsmol l^–1^) using a gravity-feed system at room temperature (20–23°C) with a flow rate of approximately 4 ml min^−1^. Time for complete bath solution exchange was approximately 5 s, but agonist onset times were dependent on position of perfusion line and cell; the rise-time of agonist-evoked whole-cell currents was < 2 s and all responses were measured at steady state. We observed that faster solution exchange rates were frequently associated with hECNs detaching from coverslips. The ‘external’ recording solution was supplemented with CNQX (5 μm), d-AP5 (50 μm), TTX (300 nm), and in the case of GABA_A_R experiments, strychnine (20 μm). Recordings were made at a holding potential of 0 mV (−14 mV when corrected for the liquid junction potential), which gave a large driving force (∼80 mV), resulting in inward flux of Cl^−^ ions. Series resistances (*R*_s_) were between 10 and 30 MΩ and compensated between 50 and 80%. Experiments were terminated if series resistance shifted more than 20%.

Before each experiment, three bath applications of a given concentration of agonist that gave equivalent current amplitudes within 15% of the initial amplitude were obtained to establish a stable response. Similarly, a response to a control concentration of agonist was applied at the end of the recording to ensure stability. Data were only taken if the amplitude of the final control response was within 15% of the initial controls. Selective agonists, antagonists and allosteric modulators were purchased either from Tocris Bioscience (Bristol, UK) or Abcam (Cambridge, UK).

### RNA-sequencing

For RNA-seq, RNA was isolated from four biological replicates using the Roche HP RNA Isolation kit according to manufacturer's instructions. Total RNA was assessed for quality (Agilent Bionalyzer) and quantity (Invitrogen Qubit) before library preparation. Illumina libraries were prepared from 1 μg of total RNA using TruSeq RNA Sample Prep Kit v2 with a 10 cycle enrichment step as per the manufacturer's recommendations. Final libraries were pooled in equimolar proportions before Illumina sequencing on a HiSeq 2500 platform using 100 base paired-end reads in rapid mode. Raw reads were processed using RTA 1.17.21.3 and Casava 1.8.2 (Illumina). Reads were mapped to the primary assembly of the human reference genome contained in Ensembl release 75. A genome index was built with Bowtie, version 1.0.0; default options; (Langmead *et al*. 2009), and then reads mapped with TopHat, version 2.0.10, (Kim *et al*. 2013); for TopHat, coverage-based search for junctions was disabled, otherwise default values were used for all options. Gene expression was then estimated with Cufflinks, version 2.2.0, (Trapnell *et al*. 2010; Roberts *et al*. 2011) using gene annotations from Ensembl release 75. Cufflinks was run in expression estimation mode only (-G flag), and corrections for multi-read mapping (-u flag) and bias (-b flag) were enabled; otherwise default values were used for all options. Estimates of GABA_A_R and GlyR subunit mRNA expression were then extracted in units of fragments per kilobase of exon per million mapped fragments, and normalised as expression relative to that of the highest expressed subunit.

### Data analysis

Recordings were low-pass filtered at 2 kHz, digitised at 10 kHz *via* a BNC-2090A (National Instruments, TX, USA) interface, and recorded to computer using the WinEDR V2.7.6 Electrophysiology Data Recorder (J. Dempster, University of Strathclyde, UK, http://spider.science.strath.ac.uk/sipbs/software_ses.htm)

Agonist concentration–response curves were fitted individually for each cell using the Hill equation:


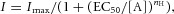


where *I* is the current response to agonist concentration [A], *n*_H_ is the Hill coefficient, *I*_max_ is the maximum current and EC_50_ is the concentration of agonist that produces a half-maximal response. Each data point was normalised to the fitted maximum of the concentration–response curve, then pooled, averaged and re-fitted again with the same equation, with the maximum and minimum for each curve being constrained to asymptote to 1 and 0, respectively (Frizelle *et al*. 2006; Wrighton *et al*. 2008).

Concentrations of antagonists required to inhibit agonist-evoked responses by 50% (IC_50_) were determined by fitting inhibition curves with the equation:


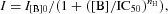


where *n*_H_ is the Hill coefficient, *I*_[B]0_ is the predicted current in the absence of antagonist and [B] is the concentration of the antagonist. Data points were again normalised to the fitted maximum, before pooling, averaging and re-fitting as described above.

Data are presented as mean ± standard error of the mean (SEM). The number of experimental replicates (cells) is denoted as ‘*n*,’ while ‘*N*’ represents number of *de novo* preparations of batches from which ‘*n*’ is obtained. Statistical analysis was conducted as described in the text with the significance levels indicated as: *P* < 0.05 (*), *P* < 0.01 (**) and *P* < 0.001 (***).

## Results

### GABA_A_ receptor characterisation

**Figure 1:**
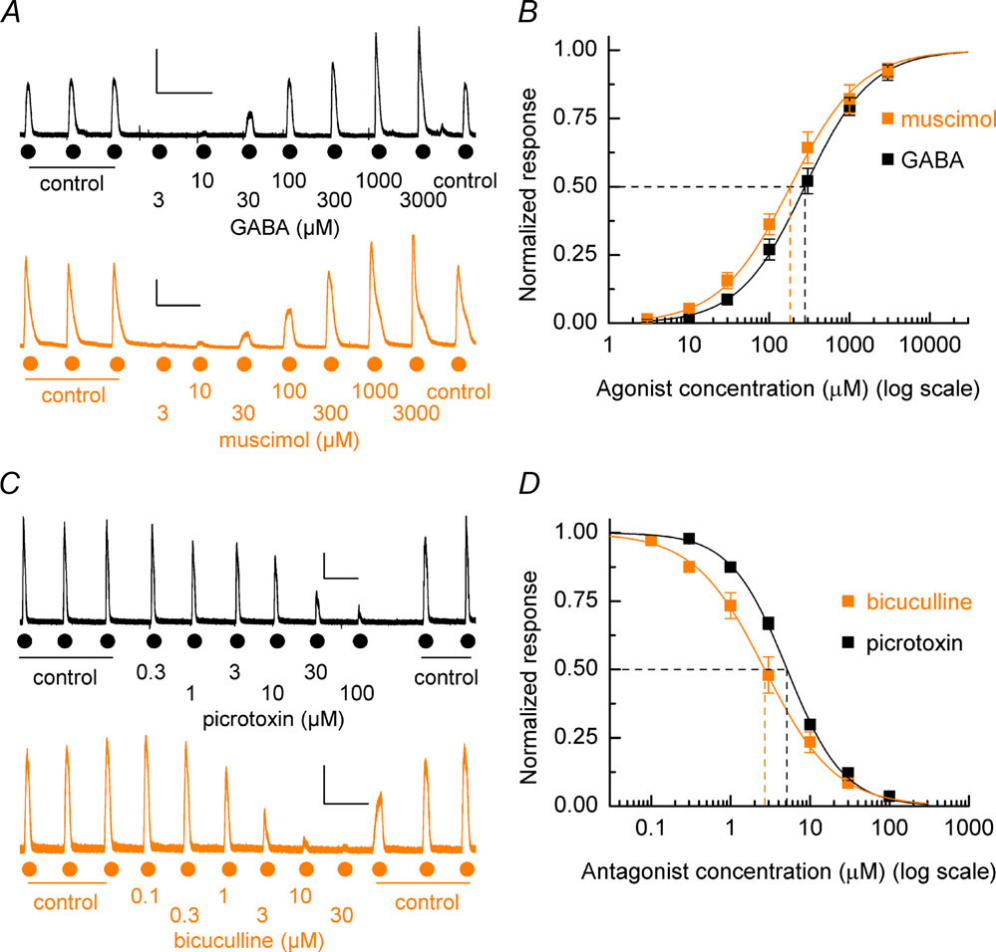
Agonist and antagonist pharmacology of hECN GABA_A_Rs *A*, representative whole-cell current recordings of GABA and muscimol concentration–response experiments. Currents were elicited by increasing concentrations of bath applications of GABA and muscimol (3 μm to 3 mm) after establishing 3 control GABA-evoked currents as indicated. Calibration bars 250 pA, 100 s. *B*, mean agonist concentration–response curves for GABA and muscimol. Mean GABA data: EC_50_ = 278 ± 11 μm, *n*_H_ = 1.05 ± 0.02, *n* = 12, *N* = 2. Mean muscimol data: EC_50_ = 182 ± 10 μm; *n*_H_ = 0.99 ± 0.02; *n* = 6, *N* = 2. *C*, example currents illustrating the inhibition of GABA-evoked responses by increasing concentrations of picrotoxin (upper panel) and bicuculline (lower panel). Calibration bars 250 pA, 100 s. *D*, mean inhibition curves for picrotoxin and bicuculline antagonism of GABA (EC_50_) evoked currents. Mean bicuculline data: IC_50_ = 2.7 ± 0.2 μm; *n*_H_ = 0.98 ± 0.03; *n* = 5, *N* = 2. Mean picrotoxin data: EC_50_ = 5.1 ± 0.2 μm; *n*_H_ = 1.22 ± 0.03; *n* = 4, *N* = 2.

The potency of GABA_A_R agonists varies considerably between GABA_A_R isoforms (Mortensen *et al*. 2011; Karim *et al*. 2013). Thus, to characterise initially the functional properties of GABA_A_Rs expressed by hECNs (28–42 DIV) differentiated from anterior neural precursors derived from H9 human embryonic stem cells (Bilican *et al*. 2014; see Methods) we conducted concentration–response experiments using GABA and the GABA_A_R-selective agonist muscimol. We previously established that hECNs robustly respond to GABA at this time point (Livesey *et al*. 2014). After establishing stable control responses to bath applications of GABA (100 μm), or muscimol (300 μm), increasing concentrations of agonist were applied sequentially to generate concentration–response curves (Fig. 1*A*). Mean EC_50_ values for GABA- and muscimol-activated currents were found to be 278 ± 11 μm (*n* = 12, *N* = 2) and 182 ± 10 μm (*n* = 6, *N* = 2), respectively (Fig. 1*B*). GABA (EC_50_)-evoked current responses were blocked by GABA_A_R antagonists bicuculline and picrotoxin (Fig. 1*C*) in a concentration-dependent manner (Fig. 1*D*) giving respective IC_50_ values of 2.7 ± 0.2 μm (*n* = 5, *N* = 2) and 5.1 ± 0.2 μm (*n* = 4, *N* = 2).

**Figure 2:**
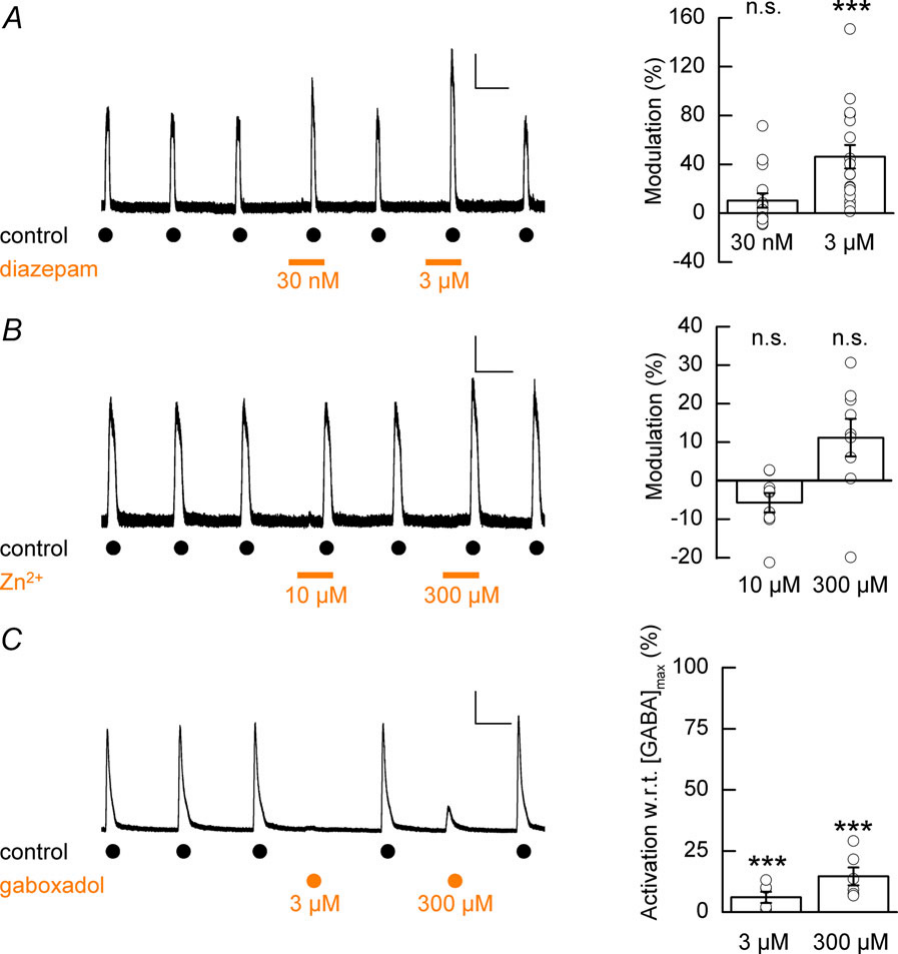
Modulation of hECN GABA_A_Rs by diazepam, Zn^2+^ and gaboxadol *A*, left panel: representative whole-cell recording depicting the co-application of diazepam (30 nm and 3 μm, as indicated by bars) to control GABA-evoked responses. *A*, right panel: modulation of GABA_A_R-mediated currents by diazepam (30 nm and 3 μm, *n* = 17, *N* = 3). Data are presented as mean percentage modulation with respect to control recordings. No difference was observed between percentage modulation and the batch from which cells were prepared. Calibration bar 50 pA, 50 s. *B*, left panel: example whole-cell recording depicting the co-application of Zn^2+^ (10 μm and 300 μm, as indicated by bars) to control GABA-evoked responses. *B*, right panel: mean percentage modulation of control GABA_A_R-mediated currents by Zn^2+^ (*n* = 9, *N* = 1). Calibration bar 100 pA, 50 s. *C*, left panel: example whole-cell recording of GABA (3 mm)-evoked currents and gaboxadol (3 μm and 300 μm)-induced currents. *C*, right panel: mean percentage gaboxadol-induced activation of GABA_A_R currents with respect to (w.r.t.) maximum GABA-evoked currents (*n* = 6–7, *N* = 1). Calibration bar 500 pA, 50 s.

We next performed a series of pharmacological assays to assess the presence of γ and/or δ subunit-containing GABA_A_Rs. Applications of γ-selective allosteric potentiator diazepam (30 nm and 3 μm) to GABA (EC_10_; 35 μm)-mediated currents potentiated the control GABA response by 10 ± 6 % (*P* = 0.1 *vs*. control) and 46 ± 10 % (*P* < 0.001 *vs*. control, Welch's *t* test, *n* = 17, *N* = 3), respectively, indicating the presence of the γ subunit (Fig. 2*A*). In contrast, applications of Zn^2+^ (10 μm and 300 μm), which selectively inhibits GABA_A_Rs composed of α and β subunits only (Draguhn *et al*. 1990), did not inhibit GABA (EC_50_)-evoked currents (10 μm, 6 ± 3 %, *P* = 0.053 *vs*. control; 300 μm, 11 ± 5 %, *P* = 0.052 *vs*. control; unpaired *t* tests; *n* = 9, *N* = 1; Fig. 2*B*). Furthermore, the potent δ-containing GABA_A_R-selective agonist gaboxadol (3 μm and 300 μm; Storustovu & Ebert, 2006) gave only nominal currents (6.0 ± 2.3% and 14.6 ± 3.7%; both data *P* < 0.001 *vs*. GABA (3 mm); unpaired *t* tests; *n* = 6–7, *N* = 1, respectively) compared to the maximum response that could be elicited by GABA (3 mm; Fig. 2*C*), confirming that a population of GABA_A_Rs that contain δ subunits is negligibly expressed. We confirmed that the low potency of GABA we observed was not a consequence of the specific culture conditions that we employed. Indeed GABA potency was not influenced by the culture of hECNs in atmospheric O_2_ 48 h prior to recording (222 ± 13 μm, *n* = 3, *N* = 1), the absence of brain-derived neurotrophic factor and glial cell-derived neurotrophic factor media supplements (222 ± 36 μm, *n* = 5, *N* = 2), or maintaining hECNs for extended (49–56 DIV) culture periods (204 ± 17 μm, *n* = 5, *N* = 2). Moreover, even for hECNs maintained for extended culture periods gaboxadol (300 μm)-evoked currents remained very low (9.7 ± 4.1 %, *n* = 4, *N* = 1) with respect to GABA-evoked currents and indicated that hECNs maintained in culture for prolonged time periods (49–56 DIV) did not begin to express a δ-containing receptor population.

**Figure 3:**
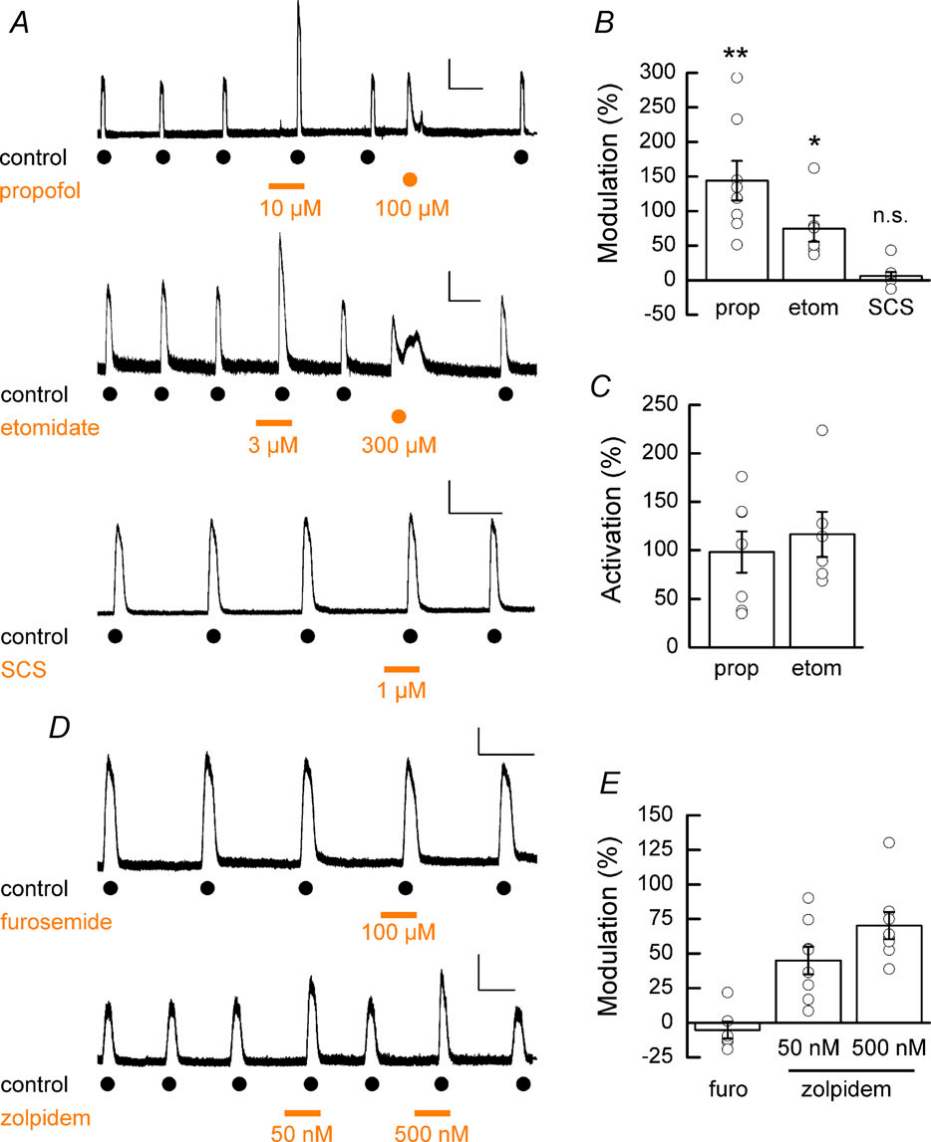
Modulation of hECN GABA_A_Rs by intravenous anesthetics, SCS, furosemide and zolipidem *A*, upper panel: example trace showing potentiation of GABA-mediated whole-cell currents and direct activation of GABA_A_Rs by propofol. *A*, middle panel: example trace showing potentiation of GABA-mediated whole-cell currents and direct activation of GABA_A_Rs by etomidate. *A*, lower panel: example trace showing lack of inhibition of GABA-mediated whole-cell currents by SCS. Calibration bars: 100 pA, 50 s (upper); 100 pA, 50 s (middle); 250 pA, 50 s (lower). *B*, mean percentage modulation GABA-induced currents by the allosteric modulators propofol (10 μm; *n* = 8, *N* = 2), etomidate (3 μm; *n* = 6, *N* = 1) and SCS (1 μm; *n* = 8, *N* = 2). *C*, mean percentage direct activation propofol and etomidate expressed with respect to control responses to GABA. *D*, upper panel: example trace showing lack of inhibition of GABA-mediated whole-cell currents by furosemide. *D*, lower panel: example trace showing potentiation of GABA-mediated whole-cell currents by zolpidem (*n* = 6–8, *N* = 2 for each condition). Calibration bars: 150 pA, 50 s (upper); 100 pA, 50 s (lower). *E*, mean percentage modulation GABA-induced currents by the allosteric modulators furosemide (100 μm) and zolpidem (50 nm and 500 nm).

The presence of β subunits in hECN GABA_A_Rs was confirmed by the potentiation by the intravenous anaesthetic propofol (10 μm) of GABA (EC_30_; 120 μm)-evoked currents which resulted in robust potentiation of the control current responses by 144 % ± 29 % (Fig. 3*A* and *B*; *P* = 0.002 *vs*. control, unpaired *t* test, *n* = 8, *N* = 2; Sanna *et al*. 1995; Hill-Venning *et al*. 1997). Furthermore, direct activation of GABA_A_Rs was observed when propofol (100 μm) was applied on its own (98 ± 21 % relative to GABA (EC_30_; 120 μm)-evoked control; *n* = 7, *N* = 2; Fig. 3*A* and *C*). The intravenous anaesthetic etomidate (3 μm), which is selective for β2/3 subunit-containing GABA_A_Rs (Hill-Venning *et al*. 1997), also potentiated GABA (EC_30_; 120 μm)-evoked currents by 75 ± 20 % (Fig. 3*A* and *B*; *P* = 0.01 *vs*. control, unpaired *t* test, *n* = 6, *N* = 1) while application on its own and at a higher concentration (300 μm) directly activated GABA_A_Rs (116 ± 23 % relative to GABA (EC_30_; 120 μm)-evoked control; *n* = 6, *N* = 1). Taken together, these data suggest the presence of a large complement of β2/3-containing GABA_A_Rs. The absence of β1-containing GABA_A_Rs was indicated by the fact that the selective inhibitor of β1-containing GABA_A_Rs, SCS (Thompson *et al*. 2004), failed to antagonise GABA (EC_30_; 120 μm)-evoked currents (Fig. 3*A* and *B*; SCS *vs*. control, *P* = 0.27 *vs*. control, unpaired *t* test, *n* = 8, *N* = 2).

As illustrated above GABA-evoked currents are potentiated by diazepam which suggests that α4 and α6 subunits are absent from the GABA_A_R population in hECNs since typically benzodiazepines are active at α1, α2, α3, or α5-containing GABA_A_Rs (Olsen & Sieghart, 2009). To rule out the possibility of the expression of α4 and α6 subunits, GABA (EC_30_; 120 μm)-elicited currents were shown to be insensitive to the α4/α6 subunit containing GABA_A_R inhibitor furosemide (100 μm; *P* = 0.43 *vs*. control, unpaired *t* test, *n* = 6, *N* = 2; Fig. 3*D* and *E*; Knoflach *et al*. 1996; Wafford *et al*. 1996). Furthermore, the observed low GABA and muscimol potencies (Fig. 1*B*) argues against the expression of α4 and α6 subunits, and also α5 subunits, which typically display high GABA potency (Mortensen *et al*. 2011; Karim *et al*. 2013). To identify the nature of the α subunit we examined the actions of zolpidem (50 nm and 500 nm), which exhibits selectivity for α1-containing GABA_A_Rs with lesser potency at α2- and α3-containing GABA_A_Rs and negligible activity at α5-containing GABA_A_Rs (Sanna *et al*. 2002). Co-application of zolpidem to GABA (EC_10_; 35 μm)-evoked currents resulted in only a mild potentiation of control currents (Fig. 3*D* and *E*; 50 nm: 46 ± 10 %; 500 nm: 70 ± 10 %, *n* = 8, *N* = 2), indicating the majority of the GABA_A_R population expressed by hECNs most likely contain α2 and/or α3 subunits.

**Figure 4:**
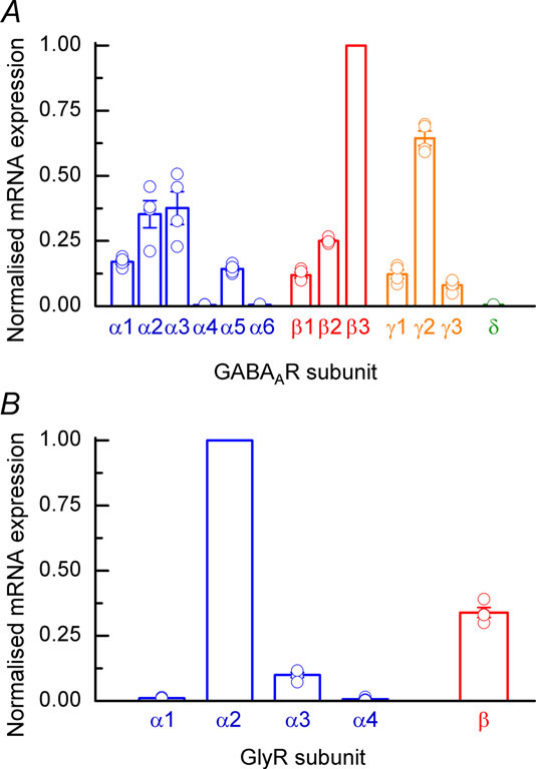
RNA-seq analysis of GABA_A_R and GlyR subunits *A*, mean human GABA_A_R subunit mRNA estimated abundances (*N* = 4) derived from RNA-seq analysis of hECNs (DIV 35). Data are normalised to the largest mRNA signal (β3 subunit). The relative expression of other GABA_A_R subunits (ε, π, θ and σ) gave signals that were considered to reflect the absence of mRNA for these subunits. *B*, RNA-seq analysis human GlyR subunits as described in *A*. Data are normalised as expression relative to α2 subunit mRNA signal.

To assess quantitatively the expression of GABA_A_R subunits we examined the relative expression of subunit mRNA transcripts via RNA-seq analysis (35 DIV). Figure 4*A* shows the relative expression of α, β and γ subunits with levels normalised to the highest expressed subunit mRNA (β3). These data are consistent with the pharmacological analysis of GABA_A_Rs expressed by hECNs described above. For α subunits, we found prominent mRNA expression of the α2 and α3 subunits and very little detection of α4 and α6 subunits, whilst the α1 and α5 subunit mRNAs were expressed to a moderate extent. The β3 subunit is prominently expressed over β1 and β2 subunits. Pharmacological data do not point to the identity of the γ subunit(s) that are functionally expressed by hECNs; however, the RNA-seq data indicate the strongest expression of the γ2 subunit mRNA. In agreement with the pharmacological analysis, levels of δ subunit mRNA expression were considered to be nominal.

### Strychnine-sensitive glycine receptor characterisation

GlyR characterisation was initially performed with RNA-seq analysis of GlyR subunit mRNA transcripts in hECNs (35 DIV; Fig. 4*B*). Both α2 and β subunits are abundantly expressed at the mRNA level, whilst α1 and α3 subunits are only nominally or weakly expressed, respectively, relative to the α2 subunit. As expected, the presence of α4 subunit mRNA was not detected given its status as a pseudogene in humans (Lynch, 2009).

**Figure 5:**
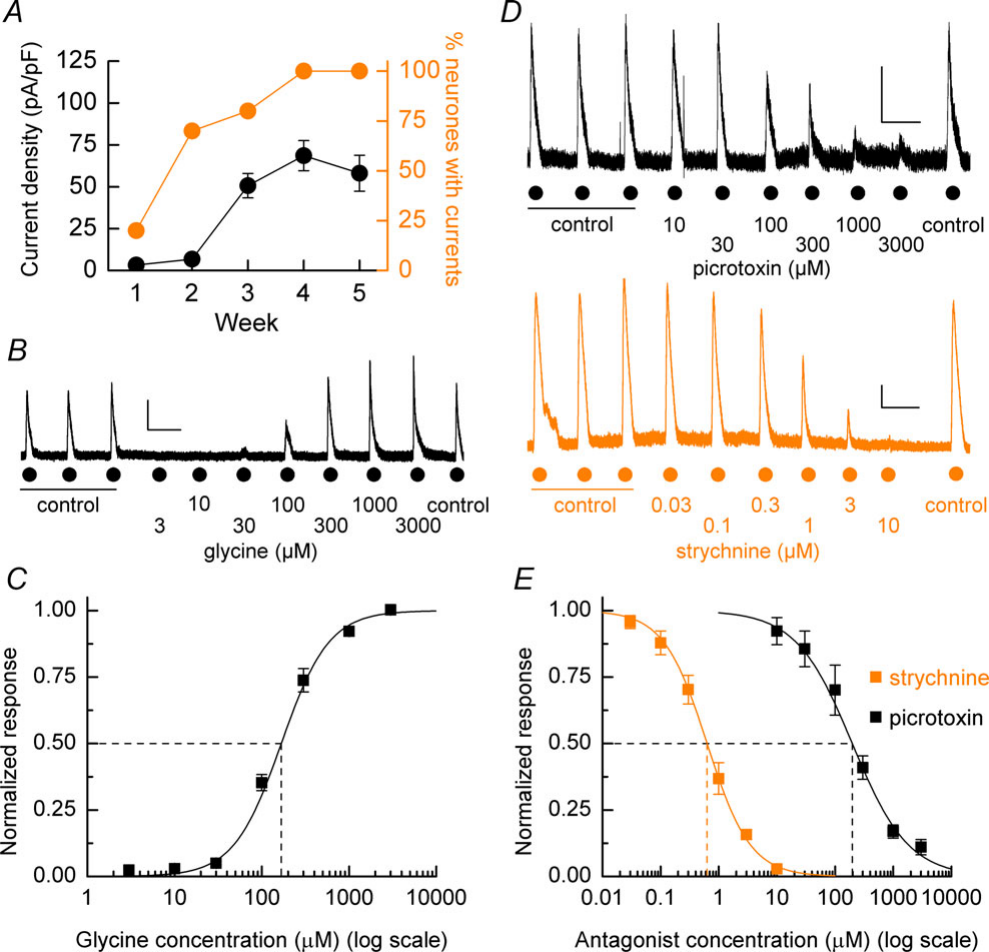
Agonist and antagonist pharmacology of hECN GlyRs *A*, weekly percentage response to bath applications of glycine and the mean glycine-mediated current density. *n* = 25–31, *N* = 3. *B*, representative whole-cell current recordings of glycine concentration–response experiments. Currents were elicited by increasing concentrations of glycine after establishment of 3 control glycine-evoked currents. Calibration bar 125 pA, 50 s. *C*, mean (± SEM) agonist concentration–response curve for glycine. Mean glycine data: EC_50_ = 167 ± 20 μm; *n*_H_ = 1.59 ± 0.1; *n* = 7, *N* = 2. *D*, upper panel: example current recording of the inhibition of glycine-evoked (500 μm) responses by increasing concentrations of picrotoxin. *D*, lower panel: strychnine inhibition of glycine-evoked (500 μm) currents amplitudes. Calibration bars 125 pA, 50 s. *E*, mean inhibition curves for picrotoxin and strychnine antagonism of glycine-evoked currents. Mean picrotoxin data: IC_50_ = 197 ± 22 μm; *n*_H_ = 0.9 ± 0.06; *n* = 5, *N* = 2. Mean strychnine data: EC_50_ = 690 ± 59 nm; *n*_H_ = 1.17 ± 0.06; *n* = 5, *N* = 2.

Functional expression of GlyRs was examined by the ability of hECNs (7–35 DIV) to respond to bath applications of glycine (500 μm). With increasing periods following differentiation the mean GlyR-mediated current density profile displays a marked increase (Fig. 5*A*; 3.3 ± 2.2 pA pF^–1^ to 49.4 ± 8.4 pA pF^–1^; *P* < 0.001, unpaired *t* test, *n* = 7, *N* = 2), indicating a strong temporal up-regulation of functional GlyRs expressed by hECNs. Furthermore by 28 DIV all cells examined gave currents (Fig. 5*A*) and in all cases examined these were blocked by the GlyR antagonist strychnine (20 μm).

The potency of glycine-evoked currents was assessed by concentration–response experiments (Fig. 5*B)*, from which a curve-fitting of mean data yielded an EC_50_ of 167 ± 20 μm (Fig. 5*C*). Glycine-evoked (500 μm) currents were blocked fully by strychnine in a concentration-dependent manner with an IC_50_ of 630 ± 59 nm (*n* = 5, *N* = 2; Fig. 5*D* and *E*). Note that an increased agonist concentration, rather than the typical EC_50_, was used to elicit suitable current responses to measure antagonist effects. The composition of the expressed GlyRs was probed using picrotoxin, which exhibits selectivity for homomeric over heteromeric GlyR forms, as the inclusion of the β subunit into the GlyR results in a reduction in sensitivity to picrotoxin (Pribilla *et al*. 1992; Wang *et al*. 2006; Lynch, 2009). Inhibition of GlyRs by picrotoxin (Fig. 5*D* and *E*) gave an IC_50_ of 197 ± 22 μm (*n* = 5, *N* = 2), indicating the low potency of this antagonist at hECN GlyRs and suggesting that the majority of these receptors are heteromeric assembles contain α and β subunits.

## Discussion

We have employed a variety of techniques to identify the principal subunit composition of ionotropic GABA_A_Rs and GlyRs expressed by hECNs. The identification of GABA_A_R subunit regulation and expression is relevant to neurodevelopment and neurological disease and thus the ability of hPSC-derived neurones to express GABA_A_Rs that reflect those seen in native neurones is essential if such *in vitro* preparations are to be used for human-specific development and disease modelling.

Our data establish that the predominant GABA_A_R α subunits expressed by hECNs (DIV 28–45) are α2 and/or α3 subunits, which is consistent with an expression profile predominantly exhibited by embryonic rodent cortical neurones (Laurie *et al*. 1992; Fritschy *et al*. 1994). Given that GABA-evoked currents were not inhibited by furosemide, hECN GABA_A_Rs are considered to lack α4 and α6 subunits. Furthermore, the mild modulatory action of zolpidem suggests the absence of the α1 subunit which is perhaps to be expected given that this subunit is associated with a more mature neuronal phenotype (Laurie *et al*. 1992; Fritschy *et al*. 1994). In agreement with our pharmacological analysis, RNA-seq also showed only moderate expression of α1 subunits together with negligible expression of both α4 and α6 subunits compared to the relative abundance of transcripts for both α2 and α3 subunits. We considered that the functional expression of the α5 subunit, which is associated with high agonist potency, was unlikely given the relatively low levels of mRNA detected and the low agonist potencies of GABA and muscimol. Indeed, low potency is indicative of GABA_A_Rs that contain either α2 or α3 subunits (Mortensen *et al*. 2011; Karim *et al*. 2013).

High expression of the GABA_A_R β3 subunit has been associated with rodent immature cortical neurones (Laurie *et al*. 1992), though the β2 subunit is often also reported to be substantially expressed in cortical neurones (Fritschy *et al*. 1994). Potentiation of GABA-evoked currents by the low concentrations of intravenous anaesthetics etomidate and propofol, direct activation by high concentrations of etomidate and propofol, a lack of SCS inhibition and a high level of mRNA expression for the β3 subunit collectively demonstrate that hECNs are likely to predominantly express β3 subunit-containing GABA_A_Rs, although a contribution of β2 to GABA_A_R stoichiometry cannot be ruled out.

The vast majority of GABA_A_Rs in the CNS are γ2 subunit containing (Olsen & Sieghart, 2009). RNA-seq data indicate that hECNs predominantly express the γ2 subunit, in agreement with the pharmacological findings that GABA-evoked currents were potentiated by γ subunit-selective diazepam. Subsets of δ subunit-containing GABA_A_Rs are selectively expressed by certain cortical adult neuronal phenotypes and importantly are commonly associated with GABA_A_R-mediated tonic inhibition (Olsen & Sieghart, 2009). Nevertheless, our data indicate that hECNs lack δ subunit-containing GABA_A_Rs as gaboxadol gave rise to only low amplitude currents compared to those seen with GABA. Furthermore, the finding that Zn^2+^ did not inhibit GABA-evoked currents is consistent with the absence of GABA_A_Rs containing *only* α/β subunits.

We have demonstrated that both RNA-seq analysis and selective GABA_A_R pharmacology converge on a predominant GABA_A_R composition of α2/3β3γ2. Such isoforms are observed in recombinant expression systems to have low agonist potency relative to other isoforms and we similarly demonstrate that GABA_A_R expressed upon hECNs exhibit relatively low agonist potency (Karim *et al*. 2013). This GABA_A_R isoform is the most likely to be widely expressed in the immature rodent cortex (Laurie *et al*. 1992; Olsen & Sieghart, 2009). Nevertheless, our data cannot rule out the presence of other GABA_A_R isoforms expressed at a low level. However, inspection of Brainspan (Atlas of the Developing Human Brain http://www.brainspan.org/rnaseq/search) indicates that the levels of mRNA we report from the RNA-seq analysis of hECNs (35 DIV) are qualitatively similar to those seen in human cortical neurones between 12 and 21 weeks post conception. Thus, hECNs provide a system to investigate the properties of human GABA_A_R pharmacology and furthermore permit investigation of the role of GABA_A_Rs in the maturing cortical neurones (Wang & Kriegstein, 2009).

In rodents, transient functional GlyR expression is a key feature of early neocortical development (Flint *et al*. 1998; Avila *et al*. 2013a). Indeed, hECNS maintained for 28–42 DIV exhibited strong responses to glycine that were blocked by the GlyR antagonist strychnine. Glycine concentration–response experiments indicated glycine potency was lower than previously reported recombinant values (Pribilla *et al*. 1992) but is generally higher than glycine potencies observed in native cortical preparations (Flint *et al*. 1998; Okabe *et al*. 2004; Kilb *et al*. 2008; but see Avila *et al*. 2013b). The reasons for these differences are unknown, but may be related to systematic differences in the solution exchange times of these studies, where slower exchange times are more likely to give shallower observed concentration–response curves. In this regard, the ability to examine deactivation kinetics of GlyRs expressed by hECNs in isolated patches using fast agonist application may yield further details of GlyR identity (Mangin *et al*. 2003; Pitt *et al*. 2008; Krashia *et al*. 2011; Marabelli *et al*. 2013).

GlyRs expressed by rodent forebrain neurones have been described as developing from an embryonic homomeric to postnatal heteromeric (β subunit-containing) composition (Lynch, 2009). To investigate the functional GlyR composition we used the antagonist picrotoxin, which inhibits homomeric over heteromeric GlyRs (Lynch, 2009). Given the observed low sensitivity of GlyRs to picrotoxin, our results suggest that the principal GlyR identity of hECNs is likely to a heteromeric α/β assembly. Pharmacological tools to identify unambiguously the nature of the α subunit within the heteromer are lacking (but see Han *et al*. 2004); however, RNA-seq analysis indicates that α2 subunit mRNA is the most abundantly expressed. As is the case for GABA_A_R subunit expression, levels of mRNA expression for GlyRs in our RNA-seq analysis are consistent with a development age of around 12–21 weeks post conception (Atlas of the Developing Human Brain http://www.brainspan.org/rnaseq/search). Finally, it is of interest to note that there is transient expression of heteromeric α2/β GlyRs by rodent Cajal–Retzius cells in early postnatal development (Okabe *et al*. 2004). This class of neurone is considered to form a significant population in our hECN cultures (Bilican *et al*. 2014) and in this respect hECNs may provide a useful human model of GlyR development.

## Additional Information

### Authors' present addresses

G. Haghi: New World Laboratories, 500 Cartier Blvd, Laval, H7V 5B7, Quebec, Canada. B. Bilican: Novartis Institutes for Biomedical Research, 100 Technology Square, Cambridge, MA 02139, USA.

## Abbreviations

d-AP5: (2*R*)-amino-5-phosphonovaleric acid
CNQX: 6-cyano-7-nitroquinoxaline-2,3-dione
DIV: days *in vitro*
GABA_A_R: γ-aminobutyric acid receptor type A
GFAP: glial fibrilary acidic protein
GlyR: glycine receptor
hECN: human excitatory cortical neurone
hPSC: human pluripotent stem cell
PCR: polymerase chain reaction
RNA-seq: RNA sequencing
VGLUT1: vesicular glutamate transporter 1
